# Supplementary material for: Empirical methods for controlling false positives and estimating confidence in ChIP-Seq peaks
Source: BMC Bioinformatics. 2008 Dec 5;9:523. doi: 10.1186/1471-2105-9-523 (PMC2628906; doi:10.1186/1471-2105-9-523)
Supplement: Additional File 1 — A variety of html documents from the USeq web site detailing the available applications, their best usage, output file type descriptions, command line menus, etc. [file 1471-2105-9-523-S1.zip › USeqUserGuides/JavaDoc.zip!/JavaDoc/edu/utah/seq/methylation454/Reference.html]

Reference


|  |  |  |
| --- | --- | --- |
| **Overview** \| **Package** \| **Class** \| **Use** \| **Tree** \| **Deprecated** \| **Index** \| **Help** | |  |
| **PREV CLASS**   **NEXT CLASS** | **FRAMES**    **NO FRAMES**     **All Classes** |  |
| SUMMARY: NESTED \| FIELD \| CONSTR \| METHOD | DETAIL: FIELD \| CONSTR \| METHOD |  |


---


## edu.utah.seq.methylation454 Class Reference

```
java.lang.Object
  edu.utah.seq.methylation454.Reference
```

---

``` public class Reference extends java.lang.Object ```

---

**Constructor Summary**

`Reference(java.lang.String line)`
  
          Alignment file line for a reference.


| **Method Summary** | |
| --- | --- |
| `static int[]` | `findCpGs(java.lang.String seq)`             Case sensitive. |
| `java.lang.String` | `getChromosome()` |
| `int[]` | `getCpGGenomicPositions()` |
| `int[]` | `getCpGPositions()` |
| `java.lang.String` | `getName()` |
| `java.lang.String` | `getSequence()` |
| `int` | `getStartPosition()` |
| `void` | `setChromosome(java.lang.String chromosome)` |
| `void` | `setName(java.lang.String name)` |
| `void` | `setStartPosition(int startPosition)` |

**Methods inherited from class java.lang.Object**

`equals, getClass, hashCode, notify, notifyAll, toString, wait, wait, wait`

**Constructor Detail**

### Reference

```
public Reference(java.lang.String line)
```

:   Alignment file line for a reference.


**Method Detail**

### findCpGs

```
public static int[] findCpGs(java.lang.String seq)
```

:   Case sensitive.

---


### getChromosome

```
public java.lang.String getChromosome()
```

---


### setChromosome

```
public void setChromosome(java.lang.String chromosome)
```

---


### getStartPosition

```
public int getStartPosition()
```

---


### setStartPosition

```
public void setStartPosition(int startPosition)
```

---


### getCpGGenomicPositions

```
public int[] getCpGGenomicPositions()
```

---


### getCpGPositions

```
public int[] getCpGPositions()
```

---


### getName

```
public java.lang.String getName()
```

---


### getSequence

```
public java.lang.String getSequence()
```

---


### setName

```
public void setName(java.lang.String name)
```


---


|  |  |  |
| --- | --- | --- |
| **Overview** \| **Package** \| **Class** \| **Use** \| **Tree** \| **Deprecated** \| **Index** \| **Help** | |  |
| **PREV CLASS**   **NEXT CLASS** | **FRAMES**    **NO FRAMES**     **All Classes** |  |
| SUMMARY: NESTED \| FIELD \| CONSTR \| METHOD | DETAIL: FIELD \| CONSTR \| METHOD |  |


---
